# Tertiary Prevention of HCC in Chronic Hepatitis B or C Infected Patients

**DOI:** 10.3390/cancers13071729

**Published:** 2021-04-06

**Authors:** Wei Teng, Yen-Chun Liu, Wen-Juei Jeng, Chien-Wei Su

**Affiliations:** 1 Department of Gastroenterology and Hepatology, Chang Gung Memorial Hospital, Linkou Medical Center College of Medicine, Chang Gung University, Taiwan NO 5, Fu-Hsing Street, Kuei Shan, Taoyuan City 333, Taiwan; tengwei@cgmh.org.tw (W.T.); b9602082@cgmh.org.tw (Y.-C.L.); 2 College of Medicine, Chang Gung University, Taoyuan City 333, Taiwan; 3 Division of Gastroenterology and Hepatology, Department of Medicine, Taipei Veterans General Hospital, Taiwan. No.201, Sec. 2, Shipai Rd., Peitou District, Taipei 11217, Taiwan; 4 Hospitalist Ward, Department of Medicine, Taipei Veterans General Hospital, Taipei 11217, Taiwan; 5 Department of Medicine, School of Medicine, National Yang-Ming University School of Medicine, Taipei 11217, Taiwan; 6 Biomedical Science and Engineering Center, National Tsing Hua University, Hsinchu 30013, Taiwan

**Keywords:** carcinogenesis, cirrhosis, direct acting antiviral agents, interferon, nucleos(t)ide analogues, sustained virologic response

## Abstract

**Simple Summary:**

Hepatocellular carcinoma (HCC) recurrence is the major obstacle concerning patients’ survival. Tertiary prevention by antiviral therapies could reduce HCC recurrence rate in both chronic hepatitis B virus (HBV) or hepatitis C virus (HCV) infected patients. In chronic hepatitis B (CHB) patients, nucleos(t)ide analogues (Nuc) provide a more effective HCC tertiary prevention effect than an interferon (IFN)-based regimen. In chronic hepatitis C (CHC) patients, the tertiary prevention effect by direct acting antiviral agents (DAAs) was reported non-inferior to that by IFN-based therapy. Chronic hepatitis C patients left untreated had the worst survival benefit as well as shorted recurrence-free interval than those treated by either type of antiviral regimen. Although the risk of HCC recurrence could only be decreased but not diminished by antiviral therapies due to host and microenvironmental factors beyond virus infection, antiviral therapy helps to preserve and improve liver function which makes multi-modality anticancer treatment feasible to improve survival.

**Abstract:**

Hepatocellular carcinoma (HCC) ranks as a leading cause of common cancer and cancer-related death. The major etiology of HCC is due to chronic hepatitis virus including HBV and HCV infections. Scheduled HCC surveillance in high risk populations improves the early detection rate and the feasibility of curative treatment. However, high HCC recurrence rate still accounts for the poor prognosis of HCC patients. In this article, we critically review the pathogenesis of viral hepatitis-related hepatocellular carcinoma and the evidence of tertiary prevention efficacy by current available antiviral treatment, and discuss the knowledge gap in viral hepatitis-related HCC tertiary prevention.

## 1. Introduction

Hepatocellular carcinoma (HCC) ranks as the seventh most common tumor and the fourth most common cause of cancer-related death worldwide [1], with the overall 5-year survival rate in the past four decades <20% globally [2]. HCC is not only a major health problem in Asian countries, where it is endemic with chronic hepatitis B virus (HBV) infection, but is also a growing issue in Western countries as a consequence of chronic hepatitis C infections. Secondary liver cirrhosis from chronic inflammation due to virus activity is the major cause of HCC [3].

The propagation of HCC surveillance programs in high risk populations including chronic hepatitis B (CHB)- or chronic hepatitis C (CHC)-infected patients increases the probability of early detection and curative management [4]. In early stage HCC patients, curative treatment including intrahepatic local treatment and surgical resection has increased the survival from 3-year 13–26% in untreated patients to 5-year 37–75% [5,6]. Even so, high HCC recurrence rate and decreased residual liver reserves in cirrhotic livers are factors in the poor survival rate of patients receiving curative HCC treatment [7,8,9,10]. However, these survival data were reported in the era before highly potent nucleos(t)ide analogues (Nuc), including entecavir (ETV) and tenofovir, for HBV viral suppression and direct acting antiviral agents (DAAs) for HCV eradication were available. This review examines the impact of advances in antiviral therapy on HCC tertiary prevention and progress towards the unmet goal of a sustained HCC cure.

### 1.1. Viral Factors Associated with HCC Recurrence: HBV

Although surgical resection or local ablation therapy provides an acceptable 5- and 10-year survival rate of 60–75% and 35–50% in patients with HBV-related HCC, the high 5-year recurrence rate, 60–80%, leads to dismal long-term outcomes [11,12,13,14,15,16]. Around 70% of recurrence occurs within 2 years of curative treatment, also defined as “early recurrence,” while recurrence after this period is defined as “late recurrence” [17]. Based on comparative genomic hybridization analysis, early recurrence is more likely to have the same clonal origin as the original tumor, indicating that it is a metastatic tumor from the original tumor, while late recurrence shows distinct clonal origins from the original tumor, implying that it might be a de novo second primary tumor [18].

**Figure 1:**
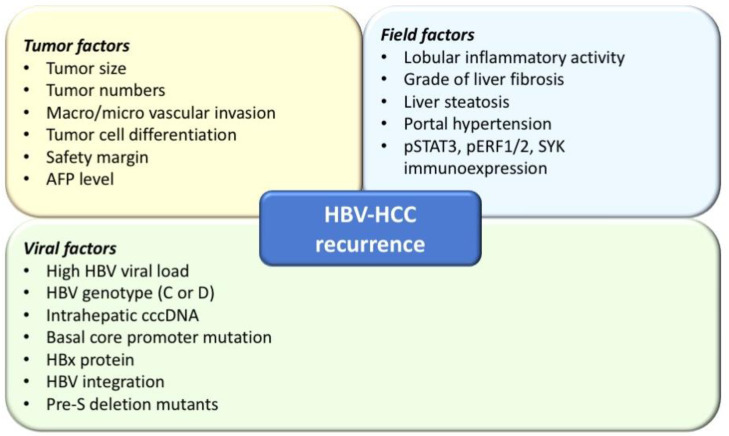
Risk factors associated with hepatocellular carcinoma (HCC) recurrence in chronic hepatitis B (CHB) patients.

Several factors associated with tumor recurrence have been identified (Figure 1), including tumor factors (tumor numbers, large tumor size, presence of microscopic vascular invasion, poor tumor cell differentiation, non-anatomical resection, narrow cut margin, high serum alpha-fetoprotein levels, etc.), field factors in the non-tumor liver parenchyma (grade of liver fibrosis and steatosis, stage of liver fibrosis, and degree of portal hypertension, as well as liver functional reserve), and HBV viral factors (including active HBV viral replication, genotypes, and mutations) [13,19,20,21,22,23,24,25,26]. Among these factors, tumor factors are associated with early HCC tumor recurrence while intrahepatic environmental factors including high serum HBV DNA levels and higher Ishak inflammation scores are predictive of late recurrence [17]. In a recent study of 645 HCC patients receiving curative surgical resection, cirrhosis, moderate/severe lobular inflammatory activity, and immunoexpression of pSTAT3, pERF1/2, and SYK proteins were predictors for late HCC recurrence [27].

In addition to high HBV viral load, HBV genotypes (C or D), presence of basal core promotor mutations, nuclear expression of HBx protein, HBV integration, and pre-S deletion mutants are associated with increased risk of HCC occurrence and recurrence in CHB patients [28,29,30,31]. HBx is a key regulatory nonstructural protein of the virus, related to host cell cycle regulation, the intersection of HBV infection, and replication and carcinogenesis [32]. The full-length HBx and HBx C-terminal truncation can activate FXR signaling, leading to carcinogenesis [33]. From a histology-based study comparing 328 HBV-associated HCC and 155 matched non-tumor tissue samples, the presence of nuclear HBx-positive HCCs indicated shorter disease free survival (DFS) (35 vs. 126 months) and higher HCC recurrence rate (58.3% vs. 39.2%, *p* = 0.015) than for those without nuclear HBx protein present [30]. In a recent study of 50 patients with HBV-related HCC (HBV-HCC), HBV integration was identified in 44 HCC tissue samples. Among the 44 patients receiving curative surgery, 10 (23.3%) had detectable plasma virus-host chimera DNA 2 months after surgery and 9 of the 10 patients experienced HCC recurrence within 1 year [31].

The deletion mutations in the pre-S region of HBV correlate with the virus escaping from host immune attack. In one mouse model study, pre-S mutants accumulated in the endoplasmic reticulum (ER), increasing ER stress and promoting hepatocyte proliferation as well as chromosome instability through the upregulation of cyclin A [34,35]. Clinical studies have also demonstrated that the presence of pre-S deletion mutants occurred in 33.8–38.9% in patients with HBV-related HCC [21,36]. Moreover, it has been shown to be an independent risk factor predicting tumor recurrence after curative surgical resection (hazard ratio (HR) 1.564, 95% confidence interval (CI): 1.057–2.314, *p* = 0.025) [21]. These findings indicate that the adverse effect of pre-S deletion mutants might be based on active HBV viral replication, in turn suggesting oncogenic risk could be reduced by antiviral therapy. In HBV-HCC patients under viral suppression, intrahepatic cccDNA level is an independent factor for intrahepatic and extrahepatic metastasis [37]. Hence, antiviral therapy may reduce but not eliminate the risk of HCC recurrence in HBV-HCC patients since current therapy can hardly deplete intrahepatic cccDNA amount.

### 1.2. Tertiary Prevention for Patients with HBV-Related HCC Following Curative Therapy: Interferon-Based Therapy

Current available antiviral treatments for chronic hepatitis B infection include interferon (IFN) and Nucs. Through a finite treatment of 48–52 weeks in duration, IFN-based regimens have immunomodulatory, antiviral, and antiproliferative effects. IFN therapy has been proven to reduce liver disease progression, incidence of HCC, and even fibrosis, especially in those with a durable response to therapy [38,39]. In a prospective randomized controlled trial (RCT) of 16 CHB-HCC patients receiving curative tumor ablation treatment, a 24-month IFN-based adjuvant therapy reduced HCC recurrence rate from 100% to 33.3% (*p* = 0.0384) [40]. However, in a larger retrospective cohort study of 568 CHB-HCC patients receiving curative surgical resection, 101 patients undergoing IFN therapy had a higher 5-year overall survival (OS) rate (69.2% vs.53.2%, *p* = 0.009) but similar 5-year recurrence-free survival (RFS) rate compared to the 467 patients without antiviral therapy (37.5% vs. 33.5%, *p* = 0.082) [41]. Moreover, another three RCTs also failed to confirm the tertiary prevention effect of IFN-based therapy in patients with HBV-related HCC who had curative resection surgery [42,43,44]. In a meta-analysis of 531 patients with HBV-HCC, IFN therapy seemed to reduce the risk of recurrence in HBV-related HCC following resection surgery, but the effect was not statistically significant (HR 0.87, 95% CI 0.70–1.09) [45]. In a recent RCT of 477 HBV-HCC patients receiving curative tumor treatment, patients who received co-administration of ETV plus Peg-IFN-α2a for 1 year and a reduction in HBsAg by >1500 IU/mL at week 48 had significantly lower recurrence and mortality rate than those who had additional IFN therapy after 1 year of Nuc treatment, underwent Nuc monotherapy, or were in the non-antiviral treated group [46]. It is still controversial whether IFN-based monotherapy has tertiary prevention effects on patients with HBV-related HCC following curative therapies, and there have not yet been the large-scale prospective randomized control studies needed to prove its efficacy [47].

### 1.3. Tertiary Prevention for Patients with HBV-Related HCC Following Curative Therapy: Nuc Therapy

Nuc therapy may achieve biochemical remission, ameliorate hepatic necroinflammation, and even regress liver fibrosis through viral suppression in CHB patients [48,49,50]. Based on several retrospective cohorts and studies using health databases, the risk of disease progression and incidence of HCC are both reduced by Nuc therapy, especially in patients with cirrhosis [51,52,53,54,55,56]. The tertiary prevention effect of Nucs in patients with HBV-HCC who receive curative tumor treatment is also demonstrated by RCTs, cohort studies, population-based studies, and meta-analysis [21,55,57,58,59,60,61,62,63,64,65,66] (Table 1). In a RCT of 200 patients with HBV-HCC undergoing surgical resection, those receiving adjuvant adefovir for 5 years had a significantly lower rate of late recurrence than those in the control group (12% vs. 29%, *p* = 0.002), although rates of early recurrence were comparable [62]. In a meta-analysis of 13 trials with 6350 patients, patients who received adefovir for HCC tertiary prevention showed a significant improvement in both OS (HR 0.56, 95% CI 0.43–0.73, *p* < 0.0001) and RFS (HR 0.66, 95% CI 0.54–0.80, *p* < 0.0001) [61]. Since Nucs are more tolerable, have fewer side effects, are applicable in patients with thrombocytopenia (commonly observed in cirrhotic patients), and are more effective for HCC tertiary prevention, applying Nucs as an adjuvant therapy is suggested in patients with HBV-HCC after curative tumor treatment.

Further, highly potent Nucs, including ETV, tenofovir disoproxil fumarate (TDF), and tenofovir alafenamide (TAF), are suitable candidates for first line regimen as they decrease the possibility of drug resistance [69]. In a study of 90 patients treated with low-potency Nucs (lamivudine, telbivudine, clevudine, or adefovir) and 256 patients receiving ETV or TDF as tertiary prevention after HCC curative treatment (radiofrequency ablation (RFA) and surgery), RFS was higher in patients using ETV or TDF than those treated with low-potency Nuc treatments (88.2 months vs. 25.1 months, *p* < 0.001) [67]. The emergence of YMDD mutation or suboptimal viral suppression during Nuc therapy was associated with increased HCC risks and shorter overall survival [67,70,71]. This suggests that highly potent Nucs give improved tertiary prevention in patients with HBV-related HCC after curative treatments.

In a Korean study of 1134 HBV-HCC patients treated with ETV or TDF after curative therapy, patients treated with TDF had a better OS and RFS than those using ETV after a propensity score matching analysis of host, viral, and tumor factors [68]. Moreover, the adjuvant TDF group had a significantly lower HCC recurrence rate for both early and late recurrence than the ETV group (early: HR 0.79, 95% CI 0.64–0.97, *p* = 0.03; late: HR 0.68, 95% CI 0.47–0.97, *p* = 0.03). However, the median follow-up duration was significantly shorter in the TDF arm than in the ETV arm (2.6 vs. 4.4 years), insufficient to compare the 5-year recurrence rate, meaning that conclusions should be drawn cautiously [72].

### 1.4. Carcinogenesis in Chronic Hepatitis C Patients

Chronic hepatitis C (CHC) infection is one of the leading risk factors for HCC, especially in Western countries, with an annual HCC risk of 2–8% in patients with cirrhosis [73,74]. Under one forecasting model, 14.4% of all HCV-infected patients are predicted to develop HCC [75]. The multi-step process of HCV-induced hepatocarcinogenesis consists of a combination of pathway alterations caused by viral factors and/or the effect of immune mediators. HCV core protein increases the levels of reactive oxygen species (ROS), leading to hepatocyte damage at both the genetic and metabolic level, eventually causing cell death. The HCV-altered environment leads to chromosomal instability and irreversible genetic changes [76,77]. HCV protein NS5A is associated with the epithelial mesenchymal transition (EMT) pathway, which promotes fibrogenesis, tumor development, and metastases [78]. In addition, somatic mutations in the telomerase reverse transcriptase (TERT) promoter that enhance TERT expression were shown to be among the earliest and most prevalent neoplastic event in HCV-related HCC (HCV-HCC) [79]. These changes promote hepatocyte neoplastic transformation and the malignant clones progression.

### 1.5. The Alteration of Host Immune System by Antiviral Therapy Differs between IFN and DAA Treatment in CHC-HCC Patients

Before DAAs were available, interferon (IFN)-α plus ribavirin was the mainstay treatment regimen for chronic hepatitis C [80]. The combination of pegylated IFN with ribavirin for 48 weeks leads to a sustained virological response (SVR) rate as high as 70%, better than IFN monotherapy [81,82]. IFN-α exerts potent antiviral activity via stimulation of IFN-stimulated genes (ISGs) and its downstream signaling pathway [83]. IFN-α can also suppress hepatocellular carcinogenesis by anti-proliferative activity including down-regulation of the Wnt pathway and extracellular signal-regulated kinase (ERK) 1/2 activation. It also causes a host immune modulation effect, anti-telomerase activity, and anti-angiogenesis activity, all of which contribute to HCC reduction [84,85,86,87,88,89].

**Figure 2:**
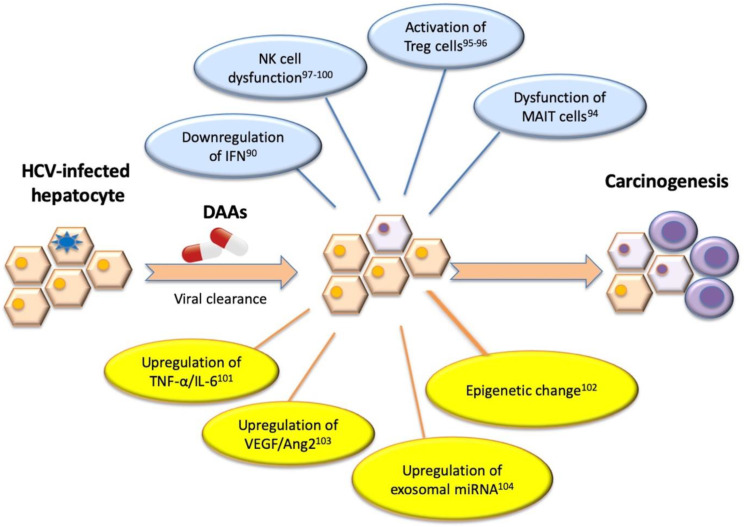
Molecular mechanism associated with HCC recurrence in chronic hepatitis C patients achieved sustained virologic response after DAA treatment. Different pathogenetic hypotheses have been postulated to investigate the development of HCC mainly based on some possible potential mechanisms including immune cell dysfunction, change in immune cytokine network, activation of angiogenesis, and epigenetic change after DAA therapy. Abbreviations: Ang2, angiopoietin-2; DAA, direct acting antiviral agents; IFN, interferon; MAIT, mucosal associated invariant T; miRNA, microRNA; NK, nature killer; TNF, tumor necrosis factor; Treg, regulatory T cell; VEGF, vascular endothelial growth factor.

In contrast, DAAs target HCV replication instead of acting on host immunomodulation. DAAs lack the IFN activation or antiproliferative effect on tumor regulation of angiogenesis, suggesting the growth of malignant cells may be tolerated [90]. The pathogenesis involved in HCC recurrence includes: 1. immune cell dysfunction; 2. change in immune cytokine network; 3. activation of angiogenesis [91]. The dysfunction of mucosal associated invariant T (MAIT) and natural killer (NK) cells, sustained immune suppression by regulatory T cells (Treg) as well as myeloid-derived suppressor cells (MDSCs), changes in the immune cytokine network (TNF-α, IL-6, IL-10, or transforming growth factor (TGF-beta)), increased expression of inhibitory molecules (programmed cell death protein 1(PD-1), cytotoxic T-Lymphocyte antigen 4 (CTLA-4)), and the activation of angiogenesis may inhibit tumor cytolysis and lead to the re-emergence of HCC during or after DAA treatment (Figure 2) [92,93,94,95,96,97,98,99,100,101]. Epigenetic changes such as H3K27ac modifications, exosomal miRNA expression of miR-211-3p, 6826-3p, 1236-3p, and 4448, and the increase of vascular endothelial growth factor (VEGF) and angiopoietin-2 levels after DAA treatment have also been reported to increase HCC recurrence risk [102,103,104].

### 1.6. The Tertiary Prevention for Patients with HCV-Related HCC Following Curative Therapy: Interferon-Based Therapy

Pegylated IFN may halt and reverse liver fibrosis after successful HCV eradication besides host immune modulation. In a large pooled study of 3010 IFN-treated CHC patients, necroinflammation improved in 39–73% of cases, while fibrosis regressed in 49% of 153 patients with baseline cirrhosis [105]. Several small-scale RCT studies and a national health insurance database study suggest the tertiary prevention effect of IFN in HCC patients receiving not only curative [106,107] but also palliative treatment [108]. In a prospective multi-center study of 105 CHC-HCC patients treated with PegIFN/RBV, patients who achieved SVR had lower HCC recurrence rate than those without (16/56 vs. 27/49, *p* < 0.01). The HCC recurrence rate was even higher in those non-SVR patients with high MHC class I polypeptide-related chain A (MICA) level >100 pg/mL [109]. In a systemic review of 13 RCTs involving 1344 patients with HCV-HCC after curative therapy, IFN reduced both early and late recurrence by 5 year follow-up (RR and P value for 1-, 2-, 3-, 4- and 5-year recurrence rate: 0.84, 0.76, 0.82, 0.79, and 0.83, respectively, all *p* < 0.01) [110]. In another meta-analysis, it was shown that SVR achieved by Peg-IFN/RBV was associated with improved overall survival and recurrence free survival in patients with HCV who have undergone resection or locoregional therapy for HCC as well as associated with a significant absolute risk reduction of recurrence (32%; 95% CI 22–42%) [111]. The tertiary prevention effect from IFN was more frequently reported as reducing late recurrence rather than early recurrence [112,113,114], and this effect was mainly attributed to patients achieving SVR [115,116].

### 1.7. The Tertiary Prevention Efficacy for Patients with HCV-Related HCC Following Curative Therapy: DAA

DAAs target various points of the HCV replication cycle, binding directly to components of the replicase complex or initiating RNA chain termination, and result in > 95% SVR in CHC patients regardless of HCV genotype, cirrhosis, or prior interferon treatment failure history within 8–24 weeks of treatment [117]. The high SVR rate and rapid elimination of chronic inflammation by DAA treatment together reduce portal hypertension, improve liver dysfunction, and regress fibrosis [118,119,120], leading to an expectation of a drastic decline in HCC recurrence. However, several studies with single-arm DAA-treated HCV-HCC patients found an increased HCC recurrence rate after antiviral treatment [121,122,123,124,125,126,127] while conflicting results have been reported in other studies, including single-arm [128,129,130] studies and studies comparing DAA treated patients to untreated [131,132,133,134,135,136,137,138,139,140] or IFN-treated groups [116,136,141,142,143,144,145]. The single-arm and comparison studies are summarized in Table 2 and the comparison studies are discussed in more detail below.

### 1.8. DAA Versus Untreated

In the prospective multi-center ANRS study, no increased HCC recurrence risk was observed in DAA treated patients when compared to untreated HCV-HCC patients [131]. In both the ANRS CO22 (HEPATHER) cohort and ANRS CO12 (CirVir) cohort, the recurrence rate was comparable between DAA-treated and untreated patients (*p* = 0.88 for overall and *p* = 0.75 for cirrhotic patients) [131]. However, an Egyptian prospective study reported a 4-fold increase in the HCC recurrence rate in DAA treated patients over untreated patients by 16 month follow-up [146]. In contrast to this, in a French study of 68 HCV-HCC patients who were all cirrhotic and receiving curative HCC treatment, the HCC recurrence rate was significantly lower among patients treated with DAAs compared with untreated patients (1.7/100 vs. 4.2/100 person-months, *p* = 0.01) [133]. In another study of 178 patients receiving DAAs after curative HCC treatment, the 2-year recurrence rate was lower in DAA treated patients than in untreated patients after adjusting host and tumor factors (2-year recurrence rate: 21.8% vs. 46.5%, Log-rank *p* < 0.01) [135]. In a recent large multicenter retrospective cohort comparing the recurrence rate between 304 patients receiving adjuvant DAA therapy and 489 patients untreated after curative HCC treatment, patients in the DAA-treated arm had an HCC recurrence rate comparable to untreated patients (HR 0.90, 95% CI 0.70–1.16) over a median follow-up duration of 10.4 months, after adjustment for study site, age, sex, Child–Pugh score, AFP level, tumor burden, and HCC treatment modality [139]. This finding has been validated by another prospective study of 163 CHC-HCC BCLC stage O/A patients with cirrhosis (HR 0.70, 95% CI: 0.44–1.13, *p* = 0.15), which applied propensity-score-matching for adjustment of the difference between DAA-treated and untreated arms including host factors, liver function, and cancer-related characteristics [140]. In addition to showing no increase in early recurrence of HCC, DAA treatment improved liver reserve and reduced mortality from hepatic decompensation, typically one of the major causes of death for CHC-HCC patients, in these patients [149].

### 1.9. DAA Versus IFN-Based Therapy

The argument that DAA is inferior to IFN treatment in tertiary prevention arises from two small studies composed of 58 and 59 CHC-HCC patients, both reporting “unexpected high” HCC recurrence rates of 27.6–28.8% within 6 months after DAA treatment with >17% of these recurrences showing more aggressive, multifocal, and infiltrative patterns [121,122]. However, these results are not significantly higher than rates reported in cirrhotic patients treated with adjuvant DAA (23.1%) [125] and in those undergoing adjuvant peg-IFN plus RBV treatment (22.2%) [150]. In a study of 443 CHC-HCC BCLC stage 0/A patients, the 2- and 5-year recurrence rate was much lower in patients receiving adjuvant IFN-based or DAA treatment (IFN vs. DAA, 2-, 5-year: 15.2% and 41.1% vs. 26.3% and 39.1%, *p* = 0.49) than in those untreated (2-, 5-year: 40.6% and 64.5%) and the recurrence was even lower in those achieving SVR regardless of antiviral regimen [116]. Similarly, several Japanese studies found no difference in recurrence pattern between IFN and DAA groups after adjustments were made for different characteristics and possible confounders [142,143,144,145]. The discrepant reports of DAA tertiary prevention effect for CHC-HCC patients may result from the differing definitions of follow-up timeframe (from HCC curative treatment or from antiviral treatment to HCC recurrence) [116,142,143,144,145].

In a recent study of 301 CHC-HCC patients with curative HCC treatment which adopted the time-varying exposure of different time frames, the incidence rate of HCC recurrence was higher in the DAA arm than in the Peg-IFN/RBV arm during anti-viral therapy (2724.4 vs. 665.8/104 person-years, log-rank *p* = 0.042) and for a 2-year period after SVR (5259.4 vs. 3277.6/104 person-years, log-rank *p* = 0.048) [148], suggesting that the tertiary prevention effect of DAA was brief during antiviral therapy and reduced/absent after the end of treatment. In contrast, a meta-analysis of 15 studies and 2352 CHC-HCC patients found that adjuvant DAA therapy was not associated with higher HCC recurrence (RR 0.62; 95% CI 0.11–3.45; *p* = 0.56) after adjustment for host and tumor factors [147]. In another systemic review and meta-analysis of nine more recent studies with a total of 1157 CHC-HCC patients, all receiving adjuvant DAA, the pooled recurrence rate was 24.4% with >77% HCC recurrence in early stages, no worse than that reported in adjuvant IFN studies and superior to that of untreated patients [136].

### 1.10. The Optimal Timing of Adjuvant DAA Treatment for Tertiary Prevention of CHC-HCC

A major concern for adjuvant DAA treatment is whether too short an interval (<4 months) between HCC curative treatment and DAA initiation will result in higher early recurrence [121,125]. Deferring adjuvant DAA therapy may allow sufficient time for immune surveillance of microscopic HCC clones as well as for the verification of HCC complete response. The sensitivity of onetime computed tomography (CT) or magnetic resonance imaging (MRI) for small HCC lesions is low, with sensitivities of only 40–50% for subcentimeter lesions and 60–70% for 1- to 2-cm lesions [151]. Given the lack of urgency for HCV therapy after HCC complete response, it therefore appears prudent to wait at least 4–6 months after HCC complete response to initiate DAA therapy, which would typically allow for 2–3 interim multiphase CT or MRI scans to confirm durable HCC response, as advised by the American Gastroenterological Association (AGA) institute [152]. Furthermore, a decrease in SVR among patients with active HCC and competing risk of HCC-related mortality has been reported in two studies [153,154] and one meta-analysis [102]. These data highlight that, when possible, treating HCV in the absence of HCC is the optimal strategy. However, the optimal period to wait before starting DAA therapy after HCC curative treatment is unknown, and whether a longer interval will increase HCC recurrence risk still awaits clarification.

### 1.11. The Role of Immune Checkpoint Inhibitors in HBV and HCV-Related HCC Patients

Immunotherapies with immune checkpoint inhibitors (ICIs), such as anti-programmed cell death-1 (PD-1) antibodies (nivolumab and pembrolizumab) [155,156,157] and anti-programmed cell death ligand 1 (PD-L1) antibodies (atezolizumab) [158] have greatly improved the survival of patients with advanced HCC due to chronic HBV or HCV infections. There was no increased incidence of major safety issues such as HBV reactivation during ICIs treatment. In a study of 60 CHB-HCC patients, no patients on antiviral therapy (regardless of HBV viral load at baseline) developed HBV reactivation, and one out of six not receiving Nucs had HBV reactivation [159]. In another large cohort of 397 patients, HBV reactivation only occurred in 2 HBsAg-positive patients (<1%) [160]. HCV reactivation is less studied in HCC patients but appears to be safe in patients with melanoma [161] and non-small cell lung cancer [162].

## 2. Conclusions and Perspective

As concerning the review papers presented in this review, we should consider some limitations. First, other adjuvant therapies for prevention of HCC recurrence, such as target therapy, immunotherapy, or chemoprevention such as metformin, statin, and so on, have not been discussed here. Second, it is difficult to compensate for the various confounding factors influencing the antiviral therapy tertiary prevention effect, including the heterogeneity in treatments for HCC, the precise definition of response, the use of antivirals, and differing follow-up timeframes.

In conclusion, although antiviral therapies can only reduce, not eliminate, the risk of HCC recurrence in patients who have already presented with intrahepatic oncogenesis, they do reduce the risk of hepatic decompensation, improve liver function, and reverse liver fibrosis in chronic HBV- or HCV-related HCC patients. By preserving or improving liver reserve, multiple and/or more radical HCC treatments may be feasible which can ultimately improve overall survival. Combination with other adjuvant therapies may also help restore host immunity or improve the intrahepatic microenvironment and thus further lower HCC recurrence in comparison to antiviral treatment alone. Further investigation of possible integrated tertiary prevention strategies is required to further reduce the HCC recurrence rate using current adjuvant antiviral therapies.

## Figures and Tables

**Table 1:** Characteristics of large-scale studies reporting hepatitis B virus (HBV)-related HCC recurrence rates after anti-viral therapy.

Author/Year	Patients No.		HCC Tx	F/U(Median)	HCC Recurrence Rate		HR	Ref.
**IFN vs. Control**								
	**IFN**	**Control**			**IFN**	**Control**		
Sun 2006 (RCT)	118	118	Resection	36.5 months	Median RFS: 31.2 (95% CI: 14.8–47.7) months	Median RFS: 17.7 (95% CI: 9.2–26.3) months	*p* = 0.1425	[42]
Lo 2007 (RCT)	40 (35 HBV)	40 (37 HBV)	Resection	5 years	5-year recurrence: 21/40 (52.5%)	5-year recurrence: 22/40 (55%)	*p* = 0.311	[43]
Qu 2010	101	467	Resection	53.3 months	year RFS: 86.1% 5-year RFS: 37.5%	year RFS: 73.9% 5-year RFS: 33.5%	0.786 (95% CI: 0.597–1.035), *p* = 0.086	[41]
Chen 2012 (RCT)	133 (106 HBV)	135 (109 HBV)	Resection	63.8 months	year RFS: 71.7% 5-year RFS: 41.7%	year RFS: 75.1% 5-year RFS: 41.3%	*p* = 0.766	[44]
Sun 2014 (meta-analysis)	264	267	Resection	NR	NR	NR	0.87 (95% CI: 0.70–1.09), *p* = 0.23	[45]
**NA vs. Control**								
	**NA**	**Control**			**NA**	**Control**		
Chan 2011	42 (38: LAM;4: ETV)	94	Resection	NR	year RFS: 66.5% 5-year RFS: 51.4%	year RFS: 48.9% 5-year RFS: 33.8%	*p* = 0.05	[57]
Wu 2012 (PSM)	518	4051	Resection	NA: 2.18 (IQR 1.21–3.69) years Control: 1.57 (IQR:0.77–3.15) years	6-year recurrence rate: 45.6% (95%CI: 36.5–54.6%)	6-year recurrence rate: 54.6% (95% CI: 52.5–56.6%)	0.66 (95% CI: 0.55–0.81), *p* < 0.001	[58]
Ke 2013 (PSM)	141 (LAM)	337	Resection	24 months	year RFS: 73.1% 5-year RFS: 44.5%	year RFS: 68.8% 5-year RFS: 43.0%	*p* = 0.503	[59]
Yin 2013 (RCT)	81 (LAM)	82	Resection	39.9 (IQR: 27.3–47.8) months	2-year RFS: 55.6% 4-year RFS: 37.3%	2-year RFS: 19.5% 4-year RFS: 12.1%	*p* < 0.001	[60]
Su 2013	62	271	Resection	45.9 (IQR: 22.4–78.9) months	year RFS: 90.2% 5-year RFS: 57.5%	year RFS :63.6% 5-year RFS :34.1%	2.296 (95%CI:1.451–3.632), *p* < 0.001	[21]
Sun 2014 (meta-analysis)	1194 (LAM)	5052	Resection; RFA	NR	NR	NR	0.66 (95% CI: 0.54–0.80), *p* < 0.0001	[61]
Huang 2015 (RCT)	100 (ADV)	100	Resection	60 (range: 4–70) months	year RFS: 85.0% 5-year RFS: 46.1%	year RFS: 84.0% 5-year RFS: 27.1%	0.651 (95% CI: 0.451–0.938), *p* = 0.021	[62]
Chong 2015	254 (ETV: 61.0%; LAM: 30.3%; ADV: 5.5%)	150	Resection	52.4 months	year RFS: 74.8% 5-year RFS: 44.7%	11-year RFS: 61.1% 5-year RFS: 38.1%	*p* = 0.166	[63]
Lee 2016 (PSM)	133	266	RFA	0.69 (95% CI: 0.50–0.95), *p* = 0.02	2-year recurrence rate: 41.8% (95%CI: 32.9–50.6%)	2-year recurrence rate: 54.3% (95%CI: 48.0–60.6%)		[64]
Wong 2016	968	1230	Resection; RFA; TACE;	2.8 (IQR: 1.4–4.9) years	Incidence of recurrence: 10.7 (95% CI: 9.3–12.2) per 100 person-years	Incidence of recurrence: 16.6 (95% CI: 15.1–18.2) per 100 person-years	0.63 (95% CI: 0.49–0.80), *p <* 0.001	[55]
Chen 2017 (meta-analysis)	2546	6463	Resection	NR	NR	NR	0.68 (95% CI: 0.51–0.67, *p*< 0.001)	[65]
**NA vs. NA**								
	**NA**	**NA**			**NA**	**NA**		
Cho 2018 (IPDW)	High-potency NA: 256	Low-potency NA: 90	Resection; RFA	53.6 months	High-potency NA Median RFS: 88.2 (IQR: 27.0–103.6) months	Low-potency NA Median RFS: 25.1 (IQR: 9.7–61.5) months	0.470 (95% CI: 0.338–0.652), *p* < 0.001	[67]
Choi 2020 (PSM)	ETV: 567	TDF: 567	Resection	ETV: 4.4 years TDF: 2.6 years	ETV 3-year RFS: 64.1%	TDF 3-year RFS: 73.2%	0.82 (95% CI: 0.68–0.98), *p* = 0.03	[68]

Abbreviations: F/U: follow-up duration; HBV, hepatitis B virus; HCC, hepatocellular carcinoma; HR, hazard ratio; IFN, interferon; RCT, randomized control trial; RFS, recurrence-free survival; CI, confidence interval; NR, not reported; LAM, lamivudine; PSM, propensity score matching; IQR, interquartile range; Ref: reference; RFA, radiofrequency ablation; ADV, adefovir; ETV, entecavir; TACE, transarterial chemoembolization; IPDW, inverse probability-of-treatment weighting; TDF: tenofovir disoproxil fumarate.

**Table 2:** Characteristics of studies reporting HCC recurrence rates after anti-viral therapy in chronic hepatitis C patients.

Author/Year	Patients No.		HCC Tx	Start of F/U, Median F/U		HCC Recurrence Rate		HR	Ref.
**DAA Alone**									
Reig 2016	58		Resection RFA TACE	HCV Tx, 3.5 months		27.6%		-	[121]
Conti 2016	59		Resection RFA TACE	HCV Tx, 5.5 months		28.8%		-	[122]
Cabibbo 2017	143		Resection RFA TACE	HCV Tx, 8.7 months		1 year: 26.6%		-	[124]
Bielen 2017	41		Resection RFA TACE	HCV Tx, 6 months		0.5 year: 14.6%		-	[128]
Ogawa 2018	152		Resection RFA TACE RT	HCV Tx, 17 months		1 year: Non-cirrhosis: 6.5% Cirrhosis: 23.1%		-	[125]
Calleja 2017	70		NR	HCV Tx, 12 months		1 year: 30.0%		-	[126]
Cheung 2016	29		NR	HCV Tx, 15 months		0.5 year:6.9%		-	[129]
Yoshimasu 2019	23		NR	HCV Tx, 21 months		1 year: 13%		-	[130]
Nakano 2019	459		Resection RFA	HCV Tx, 29.4 months		1 year: 27.1%		-	[127]
**DAA vs. Untreated**									
	**DAA**	**Untreated**				**DAA**	**Untreated**		
ANRS 2016 (CO22 HEPATHER)	189	78	NR	HCV Tx DAA: 20 months Untreated: 26 months		0.73 vs. 0.66/100 person-months (aHR: 1.04, 95% CI: 0.53–2.07)		0.88	[131]
ANRS 2016 (CO12 CirVir)	13	66	Resection RFA	HCV Tx, 21.3 months		1.11 vs. 1.73/100 person-months (aHR: 0.40, 95% CI: 0.05–3.03)		0.75	[131]
Kassas 2017 (IPTW)	53	63	Resection RFA	HCC Tx, DAA: 16 months Untreated: 23 months		37.7%	25.4%	<0.01	[146]
Virlogeux 2017	23	45	Resection RFA TACE	HCC Tx, DAA: 17 months Untreated: 10 months		1.7 vs. 4.2/100 person-months (aHR: 0.24, 95% CI: 0.10–0.55)		0.01	[133]
Ikeda 2017 (PSM)	89	89	Resection RFA TACE	HCV Tx, 20.7 months		2 years: 21.8%	2 years: 46.5%	<0.01	[135]
Huang 2018 (IPTW)	62	87	Resection RFA TACE	HCC Tx DAA: 31 months Untreated: 22 months		1 year: 47.0%	1 year: 49.8%	0.93	[138]
Singal 2019 (PSM)	304	489	Resection RFA TACE TARE/RT	HCC Tx, 10.4 months		aHR: 0.91, 95%CI: 0.69–1.19		>0.05	[139]
Cabibbo 2019 (PSM)	102	102	Resection RFA	HCV Tx, DAA: 21 months Untreated: 18 months		1 year: 15% 2 years: 27%	1 year: 20% 2 years: 40%	0.15	[140]
**IFN vs. DAA**									
	**IFN**	**DAA**				**IFN**	**DAA**		
Waziry 2017 (meta-analysis)	1485	867	NR	HCC Tx, IFN: 60 months DAA: 15.6 months		9.2 vs. 12.2/100 person-years (RR 0.62; 95%CI 0.11–3.45)		0.56	[147]
Petta 2017	57	58	Resection RFA	HCC Tx, IFN: 34 months DAA: 18 months		2 years:15.2%	2 years: 26.3%	0.49	[116]
Nagata 2017* (PSM)	22	22	Resection RFA	HCC Tx, IFN: 74 months DAA: 27 months		5 years: 54.2%	5 years: 45.1%	0.54	[143]
Mashiba 2018* (PSM)	56	56	NR	HCV Tx, IFN: 25.5 months DAA: 7.7 months		NR	NR	0.21	[145]
Kinoshita 2018* (PSM)	61	61	RFA	HCV Tx, IFN: 86.4 months DAA: 21.6 months		2 years: 61%	2 years: 60%	0.43	[142]
Nagaoki 2018 (PSM)	32	32	Resection RFA RT	HCC Tx, IFN: 63.6 months DAA: 33.6 months		1 year: 0% 3 years: 34%	1 year: 5% 3 years: 26%	0.36	[144]
Teng 2019 (PSM)	50	50	Resection RFA TACE	HCV Tx, IFN: 74.4 months DAA: 30.0 months		1 year: 22% 2 years: 48%	1 year: 48% 2 years: 58%	0.04	[148]

IFN-based therapy included PegIFN, RBV and simeprevir (SMV) or telaprevir (TVR). Abbreviations: DAA, direct acting antiviral agents; HCC, F/U: follow-up duration; hepatocellular carcinoma; IFN, interferon; IPTW, inverse probability of treatment weighting; LT, liver transplantation; NR, not reported; PSM, propensity score matching; Ref: reference; RFA, radiofrequency ablation; RT, radiation therapy; TACE, transarterial chemoembolization.

## Data Availability

Not applicable.

## Abbreviations

ADV	adefovir
AFP	Alpha-fetoprotein
BCLC	Barcelona Clinic Liver Cancer classification
CHB	chronic hepatitis B
CHC	chronic hepatitis C
CI	confidence interval
CTLA-4	cytotoxic T-Lymphocyte antigen 4
DAA	direct acting antiviral agents
DFS	disease free survival
EMT	epithelial mesenchymal transition
ER	endoplasmic reticulum
ERK	extracellular signal-regulated kinase
ETV	entecavir
FXR	Farnesoid X receptor
HBsAg	hepatitis B surface antigen
HBV	hepatitis B virus
HBV-HCC	HBV related HCC
HCC	hepatocellular carcinoma
HCV	hepatitis C virus
HCV-HCC	HCV related HCC
HR	hazard ratio
IFN	interferon
LAM	lamivudine
MAIT	mucosal associated invariant T
NK	natural killer cell
Nuc	nucleos(t)ide analogues
OS	overall survival
PD-1	programmed cell death protein 1
Peg-IFN	pegylated interferon
RCT	randomized control trial
RFA	radiofrequency ablation
RFS	recurrence-free survival
ROS	reactive oxygen species
SVR	sustained virologic response
TAF	tenofovir alafenamide
TDF	tenofovir disoproxil fumarate
TERT	telomerase reverse transcriptase
TDF-β	transforming growth factor
Treg	regulatory T cell.
